# Post-Genomics and Vaccine Improvement for *Leishmania*

**DOI:** 10.3389/fmicb.2016.00467

**Published:** 2016-04-06

**Authors:** Negar Seyed, Tahereh Taheri, Sima Rafati

**Affiliations:** Department of Immunotherapy and Leishmania Vaccine Research, Pasteur Institute of IranTehran, Iran

**Keywords:** *Leishmania*, reverse vaccinology

## Abstract

Leishmaniasis is a parasitic disease that primarily affects Asia, Africa, South America, and the Mediterranean basin. Despite extensive efforts to develop an effective prophylactic vaccine, no promising vaccine is available yet. However, recent advancements in computational vaccinology on the one hand and genome sequencing approaches on the other have generated new hopes in vaccine development. Computational genome mining for new vaccine candidates is known as reverse vaccinology and is believed to further extend the current list of *Leishmania* vaccine candidates. Reverse vaccinology can also reduce the intrinsic risks associated with live attenuated vaccines. Individual epitopes arranged in tandem as polytopes are also a possible outcome of reverse genome mining. Here, we will briefly compare reverse vaccinology with conventional vaccinology in respect to *Leishmania* vaccine, and we will discuss how it influences the aforementioned topics. We will also introduce new *in vivo* models that will bridge the gap between human and laboratory animal models in future studies.

## Leishmaniasis: The Problem

Leishmaniasis is caused by flagellated protozoan parasites in the *Leishmania* genus. The parasite’s life cycle includes two developmental stages, that is, the flagellated, motile “promastigote” and the non-motile “amastigote.” The amastigote resides and propagates within phagolysosomal vesicles of the host’s macrophages. Different sandfly species from the genera *Phlebotomus* or *Lutzomyia* transmit the parasite to human. Factors such as the parasite number, species and site of invasion, sandfly saliva, host-derived factors affecting immune-competency and the host–parasite interaction determine the severity of disease (Rodrigues et al., 2014).

Cutaneous leishmaniasis (CL) is a self-limiting infection and most often heals without any intervention. Efficient cellular immune responses control the parasite burden and amelioration. However, healing might proceed very slowly, lasting for months and eventually ending in disfiguring scars. Ulcerated lesions do not always heal despite conventional treatments (Reithinger et al., 2007). By contrast, diffuse cutaneous leishmaniasis (DCL), which is caused by *Leishmania mexicana* complexes in Brazil and Venezuela, is distinguished by producing multiple parasite-filled nodules all over the body. These nodules are not self-limiting, and they heal roughly without intervention. Skin tests with parasite proteins turn out negative, which is a direct manifestation of sub-optimal cellular responses (Convit et al., 1962). Mucocutaneous Leishmaniasis (MCL) is also a non-healing problem that is secondary to cutaneous infection with specific parasite species (especially *L. braziliensis*). Parasite invasion from the skin into the nasopharyngeal mucosa causes vast tissue destruction with irreversible disfiguration. A failure in the proper immune response regulation is responsible for the presence of few or no parasites at the lesion site (Faria et al., 2005).

There are 500,000 new cases per year of Visceral Leishmaniasis (VL), and it is fatal if left untreated, especially in children. *Leishmania* co-infection with HIV has further increased mortality rates. Immune suppression concomitant with the systemic dissemination of the parasite into visceral organs debilitates patient because of the severe internal bleeding and anemia. In South Asia and East Africa, anthroponotic VL is caused by *L. donovani*. In the Mediterranean basin, Central and South America, zoonotic disease is caused by *L. infantum*, with dogs as the primary reservoirs (Chappuis et al., 2007). Post-kala-azar Dermal Leishmaniasis (PKDL) is a complication of VL that appears as dermal nodules a few years later in VL-recovered patients in India, Nepal, and Sudan. The nodules from these patients are full of parasites, and as is the case for DCL, these nodules are very important for the transmission of the disease (Zijlstra et al., 2003).

The latest epidemiological data show that leishmaniasis is a serious global problem (Alvar et al., 2012). Despite all efforts to control it, the incidence of this disease is rising primarily because of urbanization, migration, drug resistance, and co-infection with the HIV virus (Okwor and Uzonna, 2013). The current form of control relies on chemotherapy to alleviate the disease and on vector control to reduce transmission. Although a few therapeutic chemicals are now available, including antimonials, amphotericin-B (as deoxycholate or in liposomal form), paromomycin and miltefosine, some problems such as high toxicity, variable efficacy, inconvenient treatment schedules, costs and above all, drug resistance, still remain to be addressed (Croft and Olliaro, 2011). Vector control is also a difficult task because sandflies are adapted to many different micro-landscapes (Kishore et al., 2006). Therefore, an efficient prophylactic vaccine is desperately needed in addition to new drug development. Three different generations of vaccines besides leishmanization have been the subjects of massive investigations. Among the options, live attenuated and multi-subunit vaccines are more attractive (Alvar et al., 2013; Mutiso et al., 2013).

## Immune Correlates of the Disease: CD4^+^ and CD8^+^ T Cells and Regulation

The characterization of the immune response in murine CL models that were infected with *L. major* has thus far answered some questions about susceptibility or resistance to *Leishmania* infection. *Leishmania* parasites are obligatory intracellular microorganisms. Amastigotes are sensitive to toxic oxygen and nitrogen metabolites of activated macrophages. In murine CL (C57BL/6 model), a Th1-mediated immune response by CD4^+^ T cells potentially activates macrophages primarily through IFN-γ production (Belkaid et al., 2000). Experiments by the Darrah group showed that the degree of protection against *L. major* after a needle challenge in vaccinated C57BL/6 mice depends on the frequency at which multifunctional CD4^+^ T cells are capable of simultaneously producing IFN-γ, TNF and IL-2 (Darrah et al., 2007). However, (Peters et al., 2014) showed that CD44^+^CD62L^-^T-bet^+^Ly6C^+^ T- effector cells that are short-lived in the absence of infection and produce only IFN-γ play the key role in immunity against secondary infection by sandfly challenge. Persistent parasites after healing of primary infection are responsible for induction of these effector cells that are rapidly recruited to infection site early after secondary challenge (Peters et al., 2014). The persistent production of IL-12 by dendritic cells during active infection is indispensable for the polarization and maintenance of the Th1 response (Park et al., 2000). However, the predominance of anti-inflammatory Th2 cytokines such as IL-4, IL-5, and IL-13 suppress efficient Th1 polarization and macrophage activation, thereby enhancing disease progression (Liu and Uzonna, 2012).

Although leishmaniasis is an intracellular infection, the contribution of CD8^+^ T-cells as immune correlates of the disease upon primary infection remained to be addressed (Wang et al., 1993; Huber et al., 1998) until the data from a low-dose experimental challenge in both Balb/c and C57BL/6 mice were extrapolated. The data from Balb/c mice that were infected by a low-dose challenge were controversially CD8^+^ T-cell dependent, but these mice were able to elevate the Th1-type immune response and control the primary and secondary infections (Doherty and Coffman, 1996; Menon and Bretscher, 1996; Courret et al., 2003). However, data from C57BL/6 mice clearly indicated that CD8^+^ T-cells contribute to CL control. CD8^+^ T-cell depletion at primary infection abolished resistance in C57BL/6 mice that were infected intra-dermally with 100–1,000 metacyclic promastigotes (an approximation of a low-dose natural infection) (Belkaid et al., 2002b). Uzonna et al. (2004) further noted that the IFN-γ secreted by CD8^+^ T-cells is important for directing early Th2-type responses toward Th1 and for establishing protection, which will end in a long-term memory that protects against subsequent infections (Okwor et al., 2014). The protective function of antigen-specific CD8^+^ T cells is used not only for IFN-γ production but also for the cytolysis of infected host cells that are defective in intracellular killing. Mice that are deficient in Fas or Fas ligands cannot eliminate *L. major* despite the enhanced production of nitric oxide (Huang et al., 1998). IL-10 is an important regulatory cytokine, and it plays a key role in immune response regulations in murine CL. Different cell types are responsible for IL-10 production, including CD4^+^ CD25^+^ regulatory T cells (Belkaid et al., 2002a) and CD4^+^ CD25^-^ Foxp3^-^ cells (Anderson et al., 2007; Pagan et al., 2013). IL-10 is the most important cytokine for parasite persistence after the primary infection heals (Belkaid et al., 2001).

In human CL, a clear Th1 or Th2-polarized immune response is never observed. However, an inflammatory profile is crucial for disease control and an anti-inflammatory profile exacerbates the condition. The CD4^+^ T cells are the primary contributors to pro-inflammatory cytokine production. Inter-species differences must not be neglected. Some new world species such as *L. mexicana* and *L. amazonensis* require a more robust Th1 immune response in comparison with that of *L. major* (McMahon-Pratt and Alexander, 2004). The CD8^+^ T cells and IL-10 that are produced by different cell types are also primary factors, and they play a dual role in human CL. CD8^+^ T cells contribute to the differentiation of Th1 responses during the early events of parasite infection (Pompeu et al., 2001). After the disease is cured, the CD8^+^ T cells can produce IFN-γ and participate in the healing process (Mohajery et al., 2007). IL-10 is also produced by CL patients and is responsible for down-regulating inflammatory responses, primarily those induced by IFN-γ. The presence of regulatory T cells in lesions from CL patients has already been described (Campanelli et al., 2006). CD8^+^ T cells and anti-inflammatory IL-10 and TGF-β cytokines are also responsible for immunopathology in leishmaniasis. The highest parasite loads are found in early human CL lesions (Kumar et al., 2009). Experimentally, the peak parasite load has been observed just prior to *L. major* lesion development (Belkaid et al., 2000), which supports the idea that immune-mediated skin inflammation leads to ulceration rather than a direct tissue damaging effect from the parasites (Nylen and Eidsmo, 2012). This finding is consistent with findings on chronic *Leishmania* infections such as MCL, DCL, and PKDL. MCL (Faria et al., 2009; Novais et al., 2013; Santos Cda et al., 2013) is characterized by the immunologic hyperactivity of CD4^+^ and CD8^+^ T cells (because of low IL-10 reactivity), tissue destruction, and a low parasite burden. DCL (Nylen and Eidsmo, 2012) and PKDL (Saha et al., 2007) are associated with suppressed immune responses from sustained IL-10/TGF-β production and high parasite loads without ulceration and tissue destruction.

The experimental VL shows almost the same results as the CL, although the same Th1/Th2 polarization is not clearly defined in experimental VL. Resistance to infection is Th1 response-dependent in the presence of IL-12. CD4^+^/IFN-γ^+^ T cells induced in liver are essential for parasite persistence in liver and resistance to VL reinfection in C57BL/6 mice model (Bunn et al., 2014). Recently Romano et al. (2015) have demonstrated a cross reactive immunity induced by CD4^+^/Ly6C^+^/IFN-γ^+^ T cells between *L. major* induced CL and *L. infantum* induced VL, again bolding the role of these cells this time in memory response against VL. However, studies using IL-4^-/-^ and IL-4R^-/-^ mice show that IL-4 signaling is also important for parasite clearance in the spleen and liver (Stäger et al., 2003). CD8^+^ T cells play a clear role in murine models of VL. They contribute to granuloma formation in the liver (to sequester the parasite inside macrophages). Their contribution is both pro-inflammatory cytokine production and the cytolysis of infected cells (Tsagozis et al., 2003). Joshi et al. (2009) showed that CD8^+^ T cells are exhausted during *L. donovani* infection in murine models, and that the PD1/PDL-1 pathway blockade restores the capacity of these cells to control the parasite load. The IL-10 is very important in experimental VL. The blockade of the IL-10/IL-10R pathway promotes parasite clearance (to near complete resolution) in experimental models of VL (Murray et al., 2002). In human VL, the Th1 response alone is insufficient for controlling the disease, and other factors also contribute to determine the disease outcome (Singh et al., 2012). CD8^+^ T cell exhaustion and dysfunction has been observed in the presence of high IL-10 (Gautam et al., 2014). IL-10 is primarily produced by cells other than CD4^+^ CD25^+^ regulatory T cells (Nylen et al., 2007).

Evidently, an immunological memory after *Leishmania* infection is achievable: the complete resolution of the disease results in lifelong protection. Healing from the primary infection both in mice and humans is followed by a chronic state of parasite persistence and is associated with a powerful cell-mediated immune response that rapidly deals with early immune-modulatory events at infected sandfly bite sites (Peters et al., 2009). Residual persistence is a critical factor, and the sterile cure fails to protect against further challenges (Belkaid et al., 2001; Uzonna et al., 2001; Zaph et al., 2004). The CD4^+^ effector memory and central memory T cells are two well-known components of immunity to reinfection (von Stebut, 2007). Effector memories (but not central memories; Zaph et al., 2004) owe their persistence to residual tissue parasites. IL-10 is required to suppress the anti-parasitic function of macrophages to maintain a small amount of parasites. Studies have shown that immunity to reinfection is compromised in IL-10^-/-^ mice (Belkaid et al., 2002a). Recently published data also attribute the establishment of an effective memory response to skin-resident memory CD4^+^ T cells (Gebhardt et al., 2013). Long after the resolution of the primary *Leishmania* infection, these T cell subsets are still detectable in skin sites far from the primary infection site. Resident memory T cells do not rely on residual parasites, produce IFN-γ upon re-stimulation and contribute to the rapid recruitment of cells back into the infection site, early after reinfection (Glennie et al., 2015). These early recruited T cells could be CD44^+^/Ly6C^+^ T- effector cells, however, this needs to be further addressed. Furthermore, primary *Leishmania* infection resolution is undoubtedly CD8^+^ T cell-dependent with respect to natural infection features. After complete resolution, CD8^+^ memory T cells are responsible for secondary infection control in addition to CD4^+^ T cells (Muller, 1992). These cells promote long-lasting protection, which is compromised in the absence of activated CD8^+^ T cells (Gurunathan et al., 2000; Mendez et al., 2001). Some controversial data has recently raised concern about the role of these cells in secondary infection after low dose or high dose challenge, but undoubtedly confirm that CD8^+^ T cells contribute to optimal primary immunity and establishment of successful memory response (Okwor et al., 2014). Our recently published data indicates that CD8^+^ T cells are very important in protection induced by a polytope DNA construct expressing individual MHC-I restricted peptides in Balb/c mice and that CD8^+^ T cell depletion clearly abrogates Th1 response deviation (Zandieh et al., 2015).

To conclude, despite all the unanswered questions about immunity to leishmaniasis, multifunctional CD4^+^ and CD8^+^ T cells are undoubtedly essential in a pro-inflammatory Th1 environment to control the disease. Thus far, all the vaccine approaches have focused on CD4^+^ T cell stimulations, neglecting the important CD8^+^ T cells. However, new advancements during the post-genomics era will improve vaccine design through multiple online immunoinformatics tools to mine whole genomes for potential candidates with both CD4^+^ and CD8^+^ T cell-stimulating capabilities, in addition to new strategies. Inborn differences within *Leishmania* species, the pertinent disease and a good understanding of the immunopathological mechanisms displayed by CD4^+^ and CD8^+^ T cells must be kept in mind when planning new therapeutic and vaccine strategies in human leishmaniasis. Recently published data reinforce the vaccine researchers to reconsider two main facts in *Leishmania* vaccine: the chronic parasitic infection after successful healing of primary infection (which has recently highlighted novel aspects of immunity to reinfection mediated by CD4^+^ T cells) and sandfly challenge infection instead of needle challenge.

## Conventional and Reverse Vaccinology Concepts

Historically, vaccination was introduced through the elegant experiment that was performed by Edward Jenner in 1801 (Henderson, 1997). However, Louis Pasteur was the one who established the principles of vaccination as “isolation, inactivation and inoculation” after the discovery of the causative agents of diseases. These rules provided a basis for conventional vaccinology and led to several effective vaccine developments against multiple pathogens. The *in vitro* killing or attenuation of the whole pathogen by physical or chemical methods and the isolation and characterization of potential immunogenic subunits of the cultured pathogen are the primary concerns of conventional approaches (Rappuoli, 2007). Fortunately, killed, live attenuated or even pathogen subunits have successfully lowered the incidence of many infectious diseases and have increased the average human life expectancy (André, 2003; Moriel et al., 2008). However, decades of experiments have shown that vaccine-prone pathogens are among those that actually do not undergo antigenic variation and are effectively cleared by antibody responses. By contrast, diseases that are caused by pathogens such as HIV and Influenza with numerous variants and those that demand cellular immune responses to be controlled, such as leishmaniasis, malaria and tuberculosis, still await effective vaccines (Rappuoli and Aderem, 2011).

In 1995, the complete genome of *Haemophilus influenzae* was published as the first entire genome sequence. Since then, 1000s of genomes have been sequenced and made available in data banks. The huge amount of data that is available from sequenced genomes could not be manually integrated into the desired data sets. The concomitant advancements in computer-based algorithms and “omics” such as proteomics, transcriptomics, immunomics, functional genomics and in systems biology have helped to extract the data from the genome and to integrate them into vaccine concepts (Rinaudo et al., 2009; Seib et al., 2009). This approach is called “reverse vaccinology” because it begins at the genome sequence (and not at the cell) to select potential vaccine candidates using computer-based high throughput screening (De Groot and Rappuoli, 2004). Reverse vaccinology has the potential to extend the number of subunit candidates of a pathogen from none or a few that were identified by conventional vaccinology to a genome-wide scale, saving time and energy. If the pathogen of interest is successfully controlled by humoral immune responses and neutralizing antibodies, the genome-wide screening could be further confined to the potential surface-exposed or secretory antigens. Otherwise, the genome-wide antigenic capacity is open to surveys for T cell epitopes as cellular immune response inducers.

## Current Status of *Leishmania* Vaccines: What We Have Gained From Conventional Vaccinology

Leishmanization has made an important contribution to *Leishmania* vaccine history. Exudates from active lesions containing live infectious parasites were directly inoculated into naive individuals (Nadim et al., 1983). Although it could be potentially protective by polarizing Th1 immune responses, leishmanization was discontinued because of serious concerns about its safety and standardization, problems that still remain to be resolved (Amini, 1991; Dunning, 2009). To follow Pasteur’s rule, killed *Leishmania* promastigotes entered the *Leishmania* vaccine field in 1940. Since then, different approaches have been examined to compensate the low immunogenicity of killed parasites such as BCG (Momeni et al., 1999) or CpG oligonucleotide (Heravi Shargh et al., 2012) supplementation. However, inconsistent clinical outcomes have raised serious questions about the protection potential in humans (Noazin et al., 2008).

Live parasites with attenuated pathogenicity were then developed to cover the inborn limitations of live infectious vaccination and killed vaccines. Live attenuated vaccines expose the recipient to the entire antigenic capacity of *Leishmania* and “pathogen-associated molecular patterns” that are necessary for the proper activation of immune responses but ideally lack pathogenicity potential (Silvestre et al., 2008). Conventional attenuation is achieved by exposure to chemicals, consecutive cultures or gamma irradiation (Alexander, 1982), all of which give rise to non-pathogenic strains with genetically undefined random mutations. Instead, targeted gene manipulation further facilitated the specific manipulation of virulence-related genes and resulted in many successful attenuated strains (Selvapandiyan et al., 2009; Dey et al., 2013). Although it is promising, the major concern about live attenuated vaccines is the risk of reversion to the wild type strain from compensatory gene expression in *Leishmania* (Spath et al., 2004). Therefore, human clinical trials still remain challenging. It is noteworthy to mention the importance of newly introduced nonpathogenic *Leishmania* species such as *Leishmania tarentolae*, which highly resemble pathogenic strains but lack virulence genes (Raymond et al., 2012). These species strongly stimulate Th1-type responses, and they are promising surrogates for live vaccines (Saljoughian et al., 2014).

To lower the safety risks of whole pathogen vaccines, sub-cellular components have attracted attention for generating subunit vaccines. Conventionally, subunits are identified by serological, biochemical, microbiological and molecular genetics approaches (Raju and Rao, 2010). After decades of investigation, almost 30 different protein subunits of *Leishmania* are labor intensively isolated, characterized and introduced as vaccine candidates. These subunits are primarily identified on the basis of their abundance (gp63) (Etges et al., 1985), by screening expression libraries with sera from infected animals (TSA) (Webb et al., 1998), by expressed sequence tag analyses of cDNA libraries (LeIF) (Almeida et al., 2004), by screening parasite fractions from sera obtained from infected humans (CPs) (Rafati et al., 2001) and by differential cloning (A2) (Charest and Matlashewski, 1994). Because resistance against *Leishmania* infection requires Th1-type cellular immunity and because the proteins evidently stimulate weak or no cellular responses, the characterized protein subunits have been formulated in various iterations. Many innovative adjuvants (Raman et al., 2012) including CpG oligonucleotides (Ramírez et al., 2013), delivery systems (Doroud and Rafati, 2012) including liposomes (Colhone et al., 2015), a combination of adjuvants and a delivery system (Das and Ali, 2014), DNA constructs (Taheri and Rafati, 2013), stand-alone versions (Iborra et al., 2004) or those with delivery systems (Doroud et al., 2011) and vectored vaccines (Griffiths and Khader, 2014) including non-pathogenic *L. tarentolae* (Zahedifard et al., 2014) have been extensively investigated to compensate for the low efficiency of the proteins. However, despite the satisfactory protection levels in animal models, no effective human vaccine has entered the market yet. Multi-subunit vaccines have been shown to be more promising (Rafati et al., 2005, 2006), and the only vaccine formulation that is now in human clinical trials is Leish-F. This vaccine is a tri-fusion protein composed of TSA, *Lm*STI1 and LeIF, which are three well-conserved *Leishmania* proteins, and this vaccine has successfully protected mice, hamsters, and rhesus macaques in MPL-SE formulation (Campos-Neto et al., 2001; Skeiky et al., 2002). Together, several clinical trials have shown that the LEISH-F1 + MPL-SE vaccine is safe and immunogenic in patients with LCL and MCL (Llanos-Cuentas et al., 2010; Chakravarty et al., 2011). LEISH-F1 was formulated with GLA-SE, a new promising adjuvant, and it has shown even better responses in comparison with those of MPL-SE (Coler et al., 2015). Other poly-protein vaccine formulation with diverse subunit candidates including CPA-CPB-A2 (Saljoughian et al., 2013; Shahbazi et al., 2015b) and A2-Kmp11-CPB-SMT (KSAC) (Goto et al., 2011), have also shown promising results in experimental models and even dogs (Shahbazi et al., 2015a).

Despite all successful protections conferred by different subunit vaccine formulations, unraveling the role of vector’s saliva in *Leishmania* infection (Gomes et al., 2012) raised a big concern: vector transmission of *Leishmania* abrogates vaccine-induced protection (Peters et al., 2009, 2012). Sandfly challenge massively recruits neutrophils to the infection site and strongly promotes “Trojan Horse” pathway but needle challenge is less reactive in respect to neutrophil recruitment and this might basically explain vaccination failure after sandfly challenge (Peters et al., 2009). To resolve this, several groups have used salivary related immunostimulatory proteins like SP15 as vaccine and have challenged either with needle and pertinent salivary gland homogenate/SGH (Katebi et al., 2015) or infected sandfly instead (Oliveira et al., 2015). Together their concept has shown promising in protection against *Leishmania* challenge. Respecting these results, combining effective vaccine candidates (could be mined out of whole genome sequence) and salivary proteins seems a better idea to further improve subunit vaccine approach (Kamhawi et al., 2014a; Zahedifard et al., 2014) which less complexes with safety concerns than the leishmanization or the live attenuated vaccines.

## How Can Reverse Vaccinology Ameliorate the Current Status of the *Leishmania* Vaccine?

Since the completion of the whole genome sequence of *Leishmania major* (Friedline reference strain) in 2005, approximately 8298 protein coding genes were identified on approximately 33 mega-base pair genomes (Ivens et al., 2005). This approach has attracted interest in relation to finding new vaccine candidates by reverse methods (Stober et al., 2006). In recent years, remarkable advancements in immunoinformatics science have improved potential immunogenic epitope selections from the genomes of various pathogens (De Groot et al., 2002; Tang et al., 2011). This *in silico* peptide mapping approach is the basis for “fishing antigens using epitopes as bait” (He et al., 2010) because it identifies highly ranked proteins with both CD4^+^ and CD8^+^ T cell-stimulating potential, thus helping to extend vaccine candidates (Paape and Aebischer, 2011; Aebischer, 2014; Singh et al., 2015). Furthermore, If we believe the concept in which “the most efficient immune response to some pathogens is derived from a number of different T cells that respond to an ensemble of pathogen-derived short peptides called epitopes (De Groot et al., 2002),” then epitope mapping can be further used to design “poly-epitopes” or “polytopes” as vaccines. Polytope ensembles are preferable surrogates for the pathogen body (always linked to pathogenicity reversion risk) because peptide epitopes from one potential protein, or the different proteins of one strain, or conserved proteins from different strains of a species could be easily assembled together. HLA-transgenic mice are now available from different companies to evaluate both epitope immunogenicity and polytope vaccine efficiency. Because they carry human HLA as their MHC background, these models are perfect surrogates for any other mice model. They help to evaluate not only the *in vivo* immunogenicity of predicted peptides that bind to human HLA (Seyed et al., 2014) but also the protective efficacy of polytopic constructs that encode multiple human-HLA-restricted epitopes. The latter is still missing in Leishmaniasis.

Reverse vaccinology could also make the live attenuated vaccine dream come true. A pathogen’s genome encodes thousands of proteins, and few are crucial for pathogenesis and virulence. The essential genes in *Leishmania* that are involved in promastigote to amastigote differentiation, amastigote survival and immune system evasion are apparently related to virulence. This complicates random gene attenuation by physical or chemical methods. Forward genetics identifies virulence-related genes beginning from a mutant or variant phenotype for further targeted gene manipulation (Beverley, 2003). This approach is a labor-intensive task for live attenuated parasite generation, especially in complex organisms such as *Leishmania.* By contrast, reverse vaccinology through comparative or subtractive genomics will cut the time to targeted live attenuated vaccine development by many folds since genomic sequences of pathogenic and non-pathogenic strains are now available. Different computational tools make it possible to find crucial gene/s through comparative analyses between different pathogenic strains (to distinguish species-specific genes and the core genome) (Peacock et al., 2007) and a subtractive analyses with non-pathogenic strains such as *L. tarentolae* (to find relevant virulence factors) (Raymond et al., 2012). These types of analyses allow for the identification of genes and proteins that are very specific for virulence (in addition, the prediction algorithms and *in silico* tools help to predict protein interactions in biological systems between the host and the invaded cell, which further ameliorates pathogen-specific drug screening) (Ali et al., 2013). Fortunately, most *Leishmania* strains are transfectable and will tolerate many genetic manipulations. Together with advanced transfection techniques, the targeted manipulation of pathogenic strains at actual virulence-related genes might further guarantee reversion failure.

## Fishing New Vaccine Candidates From the Genome by Using Epitopes as Bait

Epitopes are the smallest immune-stimulatory units of a protein that are presented by major-histocompatibility complexes. MHC molecules are among the most heterogeneous gene families in humans. Each allele specifically accepts peptides bearing compatible binding motifs with an HLA binding groove. The term “Epitope Mapping” is used to identify epitopes from a protein that bind MHC molecules with proper affinity and stimulate T cell (or B cell) responses. In general, MHC binding is a crucial determinant for T cell activation, but for CD8^+^ T cells, factors other than MHC binding are also important. Intracellular proteins are chopped into peptides by proteasomal enzymatic cleavage and are destined for the MHC-I compartment (via TAP molecules) in the endoplasmic reticulum. Classical approaches select immunogenic epitopes within pools of synthetic overlapping peptides (usually 15-mers) that stimulate T cell clones *in vitro* and/or *in vivo* (Basu et al., 2007; Das et al., 2014). The time and energy that are consumed this way because of the large number of peptides that must be evaluated could be saved by immunoinformatics. Immunoinformatics is the part of bioinformatics science that is concerned with the computational prediction of T cell (and B cell) epitopes from proteins (Tomar and De, 2010) and is powered to reduce the number of peptides that are valuable for further study. All we need are mathematical algorithms that are capable of predicting MHC binding, presentation and TCR activation. Both the sequence and structure of proteins have been considered during the development of predictive algorithms (Liao and Arthur, 2011; Resende et al., 2012). Those that are based on sequences include “motif-based methods,” “quantitative matrices” and “machine learning methods.”

Motif-based methods are relatively simple approaches that look for allele-specific binding motifs (Falk et al., 1991). Each allele has anchor positions that best fit with the anchor residues of a peptide. Thus, a peptide with preferable anchor residues is expected to be a binder. However, the identification of potential binders without preferred anchor residues has raised the possibility that not only the anchor but also the neighboring positions play considerable roles in MHC-peptide interaction. This line of reasoning led to matrix-based methods, although motif-based methods are still used. In any given matrix, each amino acid at each specific position has a defined score. The final score of each peptide sequence is then the sum or multiplication of individual scores used by the algorithm. All peptides derived from a protein with a given length are then ranked from the top-scored peptides to the last one. SYFPEITHI is among the very well-known matrix-based methods that are extensively used for *in silico* peptide prediction (Rammensee et al., 1999; Dikhit et al., 2015; Ip et al., 2015). Because epitopes that are extracted from MHC molecules are the primary contributors to matrix design, these methods are not actually able to discriminate between binders and non-binders. To find true positive epitopes, 10% of the top ranked peptides should be further evaluated *in vitro* and/or *in vivo*. However, matrix-based methods underestimate the impact of neighboring amino acids on the binding affinity of an amino acid at each position (non-linearity). Machine learning methods instead fix these drawbacks. Artificial Neural Networks (ANN), Hidden Markov Models (HMM), and Support Vector Machines (SVM) not only efficiently classify the peptide contents of a protein into binders and non-binders with high positive predictive values but also consider non-linearity using mathematical algorithms (Luo et al., 2015). Finally, structure-based algorithms predict epitopes with 3D-structural information from MHC molecules and peptides. According to *in silico* docking, peptides with a binding affinity for a given MHC are selected (Patronov and Doytchinova, 2013). Inborn limitations underlying motif-based and structural-based methods have made matrix-based and machine learning methods the first choices for *in silico* predictions (Shipo Wu et al., 2012) with multiple open access tools on the World Wide Web. Initially, users might become confused by the large amount of available software. However, it is better to keep in mind that the more algorithms that are used, the better the results (Yu et al., 2002).

Different groups have already started to mine the *Leishmania* genome for new vaccine candidates by using peptide maps of mouse MHC-I (Guerfali et al., 2009; Herrera-Najera et al., 2009) or human HLA-I (Schroeder and Aebischer, 2011; John et al., 2012; Singh et al., 2015) molecules. MHC-II epitope prediction is more difficult and less sensitive than MHC-I prediction. Therefore, there is still a lack of data about epitopes from *Leishmania* species in mouse or human HLA-class-II molecules to describe new potential protein candidates, and we need to collect this information. The primary priority in the *Leishmania* parasite is to focus on amastigote-specific proteins. Thus, proteomics or phospho-proteomics data from the amastigote stage will restrict whole genome screening to the amastigote-specific proteome (Paape and Aebischer, 2011). For CD8^+^ T cell epitope selection, some prefer to consider surface or secreted proteins (Naouar et al., 2016), but this consideration may cause potential epitopes from intracellular proteins to be overlooked. Recently published data show that epitopes from LPG-3 and *Lm*STI-1 as intra-cellular molecules can recall CD8^+^ T cell responses from CL-recovered HLA-A2^+^ individuals (Seyed et al., 2011). Therefore, genome-wide screening for novel antigens irrespective of sub-cellular localization could further extend the vaccine candidate list for the *Leishmania* parasite.

## Poly-Epitope Construct Design Based On Genome-Derived Epitopes

Multiple formulations are recommended for polytope ensembles. One is a direct inoculation of epitope mixtures. Because single peptides are weak immune-stimulators per se, robust adjuvanting systems such as cytokines, Toll-Like-Receptor ligands, CpG oligo-nucleotides or dendritic cell-based systems are needed. In addition, these peptides are at risk of degradation by endopeptidase or exopeptidase activity at the injection site and in circulation. Thus, putting them together in long peptide assemblies reduces the degradation risk but makes the synthesis and production rather difficult (Lu et al., 2004; Slingluff, 2011). Alternatively, self-adjuvanted nucleic acid constructs with remarkable potential to promote both CD4^+^ and CD8^+^ T cell responses are more attractive than peptide assemblies (Cho and Celis, 2012). Moreover, DNA prime-peptide boost (heterologous) regimens further potentiate T-cell responses (Moise et al., 2011). Polytope DNA constructs should be rationally designed by focusing on some critical points such as minimal junctional peptides, optimal proteasomal degradation for CD8^+^ T cell epitopes and secretory pathway guidance for CD4^+^ T cell epitopes.

Junctional peptides are inevitable in a “string of beads” in which T cell epitopes are in tandem. If dominant, these peptides will affect the immune response. To avoid junctional peptides, spacers such as AAA (Jafarpour et al., 2014), AAY (Huebener et al., 2008), K (Li et al., 2005) and AD (Bazhan et al., 2010) for CD8^+^ T cell epitopes and GPGPG for CD4^+^ T cell epitopes (Moise et al., 2011) are recommended. Spacers starting with “A” are more frequently used with respect to the “P1 premise.” Accordingly, the chance of proteasomal cleavage increases once the P1 amino acid next to the C-terminal peptide is alanine (Neisig et al., 1995). It is now possible to compare different possibilities for epitope arrangements with or without spacers by immunoinformatics, which can efficiently predict the cleavage sites on the polytope sequence (Seyed et al., 2014). Another important note is that endogenously synthesized proteins that are destined for proteasomal degradation are ubiquitinated only if they carry degradation signals (Mogk et al., 2007). Artificial proteins such as polytopes without internal signals are long-lived molecules with long half-lives before degradation. This tendency could be compensated by ubiquitination with a single ubiquitin molecule (76 amino acid long) that is covalently attached to a polypeptide chain. Each molecule recruits more molecules and consequently makes a poly-ubiquitinated polytope (Sharma and Madhubala, 2009). Alternatively, the N-terminal signal peptide could be used instead of ubiquitination (Eslami et al., 2012). Although the CD8^+^ T cell response has been shown to be essential in *Leishmania* clearance, polytopes aimed at CD8^+^ T cell induction remain to be addressed with regards to *Leishmania* infection. Our group has recently shown that a rationally designed DNA construct that encodes multiple CD8^+^ T cell epitopes from *Leishmania* proteins effectively stimulate cytotoxic T cells in both Balb/c and HLA transgenic C57BL/6 experimental models (Seyed et al., 2014). Others have focused on human HLA-I or –II epitope prediction from well-known vaccine candidates such as CPs, gp63, LeIF, *Lm*STI-1, KmP-11 and LPG-3 by using an immunoinformatics approach for future vaccine design (Saffari and Mohabatkar, 2009; Seyed et al., 2011; Elfaki et al., 2012; Rezvan, 2013; Agallou et al., 2014).

In addition to ubiquitination and optimal cleavage, T helpers are also a primary concern during the rational design of polytopes. T Helper-inducing peptides are necessary for CD8^+^ T cell priming. PADRE (Cong et al., 2012) and Tetanus Toxoid-derived peptides are extensively used for this purpose. These peptides are applicable to both mouse and human studies, and they induce Th1-type responses whenever permitted. In any case, CD4^+^ T cell-inducing epitopes should be destined for the excretory pathway, to enter the MHC class-II compartment. Signal peptides are efficient at this responsibility.

The HLA heterogeneity of human populations is still a remaining obstacle to surmount. Promiscuous epitopes presented by HLA super-types are a solution. Super-types are allelic groups with close but not exact binding motifs or “supermotifs” that bind a group of peptides with more or less comparable affinities. Nine different supertypes have already been characterized, and it is postulated that 3 out of 9, including A2, A3 and B7, cover more than 90% of the global population. The remaining percentage should be covered by population-specific alleles (Reche and Reinherz, 2007). Predicting promiscuous epitopes is an easy task now and immunoinformatics fulfills this job with algorithms such as NetMHCpan and NetMHCpanII for MHC-I and MHC-II, respectively. Although we are still far from an ideal polytope vaccine for human population, some researchers have studied the protective potential of epitope vaccines against *Leishmania* infectious challenges in experimental models (Spitzer et al., 1999; Sachdeva et al., 2009; Agallou et al., 2011; Kedzierska et al., 2012). Recently published data provide the proof of concept for T cell-based *Leishmania* vaccines. Das et al. (2014) have prepared DNA constructs that are enriched with CD4^+^ and CD8^+^ T cell stimulatory segments of four different proteins to minimize the HLA effect. The vaccine has been shown to be protective in a rodent model of VL (Das et al., 2014), and it is a candidate for human clinical trials (Riede et al., 2015). The hallmark of LEISHDNAVAX is that the vaccine antigens were tested with T cells from leishmaniasis-recovered individuals, and they have been shown to be immunogenic in genetically diverse human populations, starting from humans and ending in a human vaccine (Kamhawi et al., 2014b).

Not only can polytopic constructs serve as prophylactic vaccines, but they are also a promising approach for immunotherapy. Recently, Teh-Poot et al. (2015) successfully evaluated the immunotherapeutic potential of a mixture of 10 peptides in *Trypanosoma cruzi*-infected mice. The therapeutic vaccine controlled the resulting parasitemia, cardiac tissue inflammation and parasite burden (Teh-Poot et al., 2015).

## HLA Transgenic Mice Pave the Pathway “From Human to Human”

Mouse models are very well-known experimental models in *Leishmania* research. However, subtle differences in peptide presentations by mouse MHC or human HLA systems might be one possible explanation for the failure of protective vaccines in human trials. To fill this gap between experimental models and human applications, new experimental models were generated to express human HLA molecules in mice (Pascolo, 2005). In the preliminary models, the immune response was more mouse MHC and less human HLA-restricted. However, advancements in transgenesis and gene manipulation techniques further facilitated mice MHC knockout by replacing the gene with a complete human HLA sequence. In these transgenic animals, the human HLA allele is the only source of T-cell training in the thymus; thus, they are invaluable models for bridging the gap between laboratory and human field studies, especially for peptide prediction and polytope vaccine development.

Animals that express either human HLA class-I or -II or both class-I and II, are now available from different companies. These pre-clinical models have revolutionized studies from human *in silico* approaches to human *in vivo* experiments before being moved to clinical trials, that is to say, from humans to humans (Kotturi et al., 2009). Many investigators have harnessed the model’s potential for evaluating human-derived epitope-based vaccine efficacy both in tumor cell challenges (Dosset et al., 2012; Ding et al., 2013) and different infections such as Poxvirus (Moise et al., 2011), *Toxoplasma gondii* (Cong et al., 2012), *Mycobacterium tuberculosis* (Geluk et al., 2012) and *Plasmodium falciparum* (Mahajan et al., 2010). Our group has also recently shown the immunogenicity of a polytope DNA construct that encodes multiple CD8^+^ T cell-stimulating peptides in HLA-A2 transgenic mice as the first report in *Leishmania* (Seyed et al., 2014). Previously, Rezvan et al. (2012) showed the immunogenicity of HLA-I and HLA-II (Rezvan, 2013)-restricted peptides from *Leishmania*-gp63 in relevant pre-clinical models. However, data to support the protective efficacy of epitope vaccines against *Leishmania* are still missing.

## Concluding Remark

Today there are almost 30 different characterized proteins, out of at least 8000 proteins encoded in the parasite genome, for vaccine studies. This substantiates further characterization of new protein candidates both as virulence factors to generate more reliable live attenuated parasites and also as members of more effective multi-subunit vaccines to obtain better vaccine modalities. This could be achieved from mining the full sequenced genome of the *Leishmania* species now available. Hopefully massive data extrapolated from the genome will further revolutionize the future of vaccine design and drug development by unraveling the mysteries around the biology of the parasite.

## Author Contributions

All authors listed, have made substantial, direct and intellectual contribution to the work, and approved it for publication.

## Conflict of Interest Statement

The authors declare that the research was conducted in the absence of any commercial or financial relationships that could be construed as a potential conflict of interest. The reviewer SS and handling Editor declared their shared affiliation, and the handling Editor states that the process nevertheless met the standards of a fair and objective review.
